# Metabonomics of d-glucaro-1,4-lactone in preventing diethylnitrosamine-induced liver cancer in rats

**DOI:** 10.1080/13880209.2018.1525414

**Published:** 2018-12-11

**Authors:** Wenlong Yang, Guanlin Zhou, Shubing Zou, Wentao Yang, Aihong Liu, Shuilin Sun, Baogang Xie

**Affiliations:** a Department of Infectious Diseases, the Second Affiliated Hospital of Nanchang University; School of Pharmaceutical Science, Nanchang University, Nanchang, PR China;; b Department of Hepatobiliary and Pancreatic Surgery, the Second Affiliated Hospital of Nanchang University, Nanchang, PR China;; c Center of analysis and testing, Nanchang University, Nanchang, PR China

**Keywords:** Glucarate, prevention of liver cancer, 1HNMR metabonomics, serum AFP levels, amino acid metabolism, Fischer’s ratio

## Abstract

**Context:**d-Glucaro-1,4-lactone (1,4-GL) exists in many vegetables and fruits. Metabonomics has not been used to investigate the role of 1,4-GL in preventing liver cancer.

**Objective:** The pharmacological effects and metabolite alterations of 1,4-GL on the prevention of diethylnitrosamine (DEN)-induced liver cancer were investigated.

**Materials and methods:** Ten healthy Sprague–Dawley rats served as control and 46 were used to establish rat liver cancer model. ^1^HNMR-based metabonomics was used to compare the effects of oral 1,4-GL (50 mg/kg) in liver cancer rats (*n* = 26) after 10 consecutive weeks of intervention. The amino acids in rat serum were quantified by HPLC-UV, and the changes in Fischer’s ratio were calculated.

**Results:** The 20-week survival rate of DEN-induced liver cancer rats administered with oral 1,4-GL was increased from 45.0 to 70.0% with reduced carcinogenesis of the liver and significantly lowered serum α-fetoprotein level (14.28 ± 2.89 ng/mL vs. 18.56 ± 4.65 ng/mL, *p* = 0.012). The serum levels of leucine, valine, 3-hydroxybutyrate, lactate, acetate and glutamine in the DEN + 1,4-GL group returned to normal levels compared with those of the DEN group on week 20. Fischer’s ratio in the rat serum of DEN group was 1.62 ± 0.21, which was significantly lower than that in healthy rats (2.3 ± 0.12). However, Fischer’s ratio increased to 1.89 ± 0.22 in the DEN + 1,4-GL group.

**Discussion and conclusions:** 1,4-GL exerted positive effects on liver carcinogenesis in rats by pathological examination and metabonomic analysis. Its mechanism may be related to the restoration of amino acid and energy metabolism.

## Introduction

d-Glucaro-1,4-lactone (1,4-GL) naturally exists as an organic acid lactone in fruits, such as apples, grapefruits and oranges (Saluk-Juszczak 2010). Our previous study showed that the 1,4-GL content in apples ranged from 0.3 to 0.9 mg/g (Xie et al. 2016). β-Glucuronidase (β-GD), a hydrolase in intracellular lysosomes, is the byproduct of the reverse reaction of uridine diphosphate-glucuronosyltransferase (UGT) binding. This binding reaction decomposes glucuronic acid metabolites, such as steroids, environmental carcinogens and other substances. Therefore, the balance of the UGT and β-GD activities *in vivo* determines the ability of the body to metabolize and detoxify carcinogens (Heerdt et al. 1995; Walaszek et al. 1997). 1,4-GL is a specific inhibitor of β-GD in the body; it exerts a pharmacological activity that improves the body’s defence mechanisms and helps eliminate carcinogens (Heerdt et al. 1995; Bespalov and Aleksandrov 2012).

Hepatocellular carcinoma (HCC) is a disease that seriously threatens human health and is the third most malignant tumour in the world that causes death (Forner and Bruix 2012; Forner et al. 2012). Liver cancer can easily metastasize and infiltrate into tissues because it has no obvious clinical symptoms at its early stage. It is characterized by rapid development, high concealment and a high degree of malignancy, which are often observed in the late stage. With the increasing incidence of liver diseases in the world, the prevention and treatment of liver cancer have drawn significant attention (Forner and Bruix 2012). Early studies showed that continuous oral administration of 1,4-GL precursor calcium glucarate in rats significantly delayed and inhibited the initiation phase of diethylnitrosamine (DEN)-induced hepatocarcinogenesis (Oredipe et al. 1987, 1992).

The metabonomics technology has developed rapidly in recent decades. It mainly focuses on the alterations of the low-molecular-weight endogenous metabolites in the body with time, space and external disturbances (Robertson 2005). In recent years, several remarkable achievements have been made by metabonomics in disease diagnosis, pathogenesis, preclinical safety evaluation, drug discovery and development (Song et al. 2017; Zennaro et al. 2017; Dudzik et al. 2018; Yu et al. 2018). Abnormal metabolism is an important characteristic of tumours. Bile acid and amino acid (AA) metabolism, fatty acid β-oxidation and glycerophospholipid metabolism are associated with the development of liver cancer (Zhou et al. 2012; Budhu et al. 2013; Huang et al. 2013). Using the proton nuclear magnetic resonance spectroscopy (^1^HNMR)-based metabonomics, 1,4-GL was found to prevent hyperlipidaemia by significantly promoting the excretion of lactate, acetate and cholate in rats with a high-fat diet (Xie et al. 2014). To the best of our knowledge, there is no metabonomics report investigating the role of 1,4-GL in preventing liver cancer.

In this study, a DEN-induced rat liver cancer model was established. ^1^HNMR-based metabonomics technology was used to detect the changed metabolites in healthy rats and HCC rats with or without oral administration of 1,4-GL (50 mg/kg). The group differences in serum metabolic phenotypes were investigated. The branched-chain AAs (BCAAs) and aromatic AA in the serum of rats were quantified, and the changes in Fischer’s ratio after the oral administration of 1,4-GL were discussed.

## Materials and methods

### Instrument, drugs and reagents

Agilent 1260 HPLC [quadrupole gradient pump, degassing unit, ultraviolet–visible spectrophotometry (UV/Vis) detection, column oven, autosampler, chemical workstation (Agilent Technologies, Santa Clara, CA, USA) and Tianjin Bonaventure Technology Co., Ltd.] was used for amino acid analysis. 

DEN was purchased from Shanghai Macklin Biochemical Technology Co., Ltd. (Shanghai, China). 1,4-GL was purchased from Sigma (St. Louis, MO, USA). The rat α-fetoprotein (AFP) enzyme-linked immunosorbent assay (ELISA) kit was purchased from Nanjing Jiancheng Bioengineering Institute, China. D_2_O and sodium salt of (trimethylsilyl)-propionic-2,2,3,3-*d*4 acid (TSP) were purchased from Sigma (St. Louis, MO, USA). The following reagents were also used: norleucine (chromatographically pure; Tianjin Bonna-Agela Technologies Co. Ltd., China), triethylamine (chromatographically pure; Tianjin Bonaventure Technology Co., Ltd., China), phenylisothiocyanate (PITC, chromatographically pure; Tianjin Bonaventure Technology Co., Ltd., China), *n*-hexane (chromatographically pure; Hubei Duke Chemical Reagent Co., Ltd., China), AA standards (chromatographically pure,; Tianjin Bonaventure Technology Co., Ltd., China) and formic acid (Aladdin Chemical Reagent Co., Ltd., China). Methanol and acetonitrile were chromatographically pure.

### Rat grouping and dosing regimen

Fifty-six six-week-old healthy Sprague–Dawley rats that weighed ∼150 g each were selected and provided by the Department of Animal Science and Technology, Nanchang University. After three days of adaptive feeding, the rats were randomly divided into three groups according to their body weight. Before injection, the lower abdomens of the rats were sterilized twice a week with cotton balls dipped in 75% alcohol. The control group (*n* = 10) received an intraperitoneal injection of normal saline (0.5 mL/each time), twice a week. The injection method was the same as that for the DEN group. Twenty rats in the DEN group received an intraperitoneal injection of DEN (0.5 mL/each time) at a dose of 50.0 mg/kg with 10 weeks of continuous injection. For the DEN + 1,4-GL group, 26 rats were injected twice weekly with DEN and given 1,4-GL (50.0 mg/kg) by gavage (2.0 mL, injection and gavage at different time points) for 10 consecutive weeks. All surviving rats were sacrificed at week 20. Each group of animals, male and female, were marked with picric acid, numbered and cage fed with five rats. The animals were housed in approved facilities at a controlled relative humidity (50–70%) and temperature (22 ± 2 °C). Water and regular rodent chow were available *ad libitum*. This animal study was approved by the ethics committee of Nanchang University.

### Sample collection

On the 20th week, 2–3 mL of peripheral blood was collected from the orbit of the surviving rats in each group. Then the rats were sacrificed by cervical dislocation and their livers were removed immediately. The morphological changes in the liver were recorded, part of which were taken for pathological examination, and the remaining part was frozen at –40 °C. The whole blood was left at room temperature for approximately 2 h and centrifuged at 3000 rpm for 10 min, and then the isolated serum was frozen at –40 °C.

### Serum AFP content test

The AFP content of the rat serum was determined using the rat AFP ELISA kit. Specifically, frozen rat serum samples were taken and thawed at room temperature, and the serum AFP levels in the different groups were determined according to the kit’s instructions. After the addition of the terminating solution, the absorbance was measured at a wavelength of 450 nm within 10 min. The regression equation of the standard curve was calculated according to the concentration and the OD value, and the AFP content in the serum of each group was calculated.

### Pretreatment of rat serum for metabonomic analysis

The serum samples were thawed at room temperature. Afterwards, 200 μL of serum was added to 800 μL of methanol, vortexed and centrifuged at 10,000 rpm for 5 min. Subsequently, 700 μL of supernatant was collected and evaporated to dryness at 37 °C using a SpeedVac system (Hersey Instrument Co., Ltd, China). After drying, the concentrates were added to 450 μL of double distilled water, vortexed and ultrasonicated for 5 min to promote dissolution. Subsequently, the solution was centrifuged at 10,000 rpm for 5 min, and 400 μL of supernatant was collected. Afterwards, 50 μL of phosphate buffer (pH 7.4) and 50 μL of a 5 mmol/L TSP in D_2_O were added. The solution was transferred to a 5 mm NMR tube for ^1^HNMR data acquisition.

### ^1^HNMR data acquisition and treatment

Using a Bruker Avance II-600 MHz spectrometer (Bruker BioSpin Corp., Germany), the experimental temperature was set to 298 K using the NOESY pulse sequence. The pre-saturation method was used to suppress the water peak, and 64 scans were performed at a spectral width of 14 ppm to obtain a 1 D proton spectrum.

After the phase and baseline adjustment of each ^1^HNMR spectrum, the 3.36–3.37 ppm and 4.7–5.2 ppm areas were removed to eliminate the influence of the methanol and water peaks. To eliminate the analysis error caused by different concentrations among the samples, the total sum method normalization and unit variance scaling pretreatment were performed on each sample (Yu et al. 2018). The multivariate statistical analysis was then performed using SIMCA-P (version 12.0; Umetrics, Umea, Sweden), including principal component analysis (PCA) and partial least squares discriminant analysis (PLS-DA). The accuracy and predictability of the established PLS-DA model can be assessed with three parameters (i.e., R^2^X, R^2^Y and Q^2^Y). The PLS-DA model was validated by a seven-fold cross-validation method with 200 times permutation test. The metabolites contributing to the classification were then identified and validated according to the method in the literature (Yu et al. 2018).

All programs in this lab were run and analyzed on MATLAB (2012b; MathWorks, Natick, MA, USA), SIMCA-P (12.0, Umetrics AB, Sweden), and SPSS (22.0, IBM Corp., Armonk, NY, USA) platforms.

### Amino acids analysis of serum by HPLC-UV method

Serum (200 μL) was added into 800.0 μL of methanol, vortexed and centrifuged at 10,000 rpm for 5 min. The supernatant (700 μL) was collected and evaporated to dryness at 37 °C. Distilled water (200 μL) was added to reconstitute the sample together with norleucine (20 μL) as internal standard (IS) solution (1 mg/mL). Subsequently, triethylamine-acetonitrile solution (100 μL) and PITC-acetonitrile solution (100 μL) were added to each tube and kept at room temperature for 1 h. Then, *n*-hexane (800 μL) was added to each tube to extract the excess PITC. The lower solution (PITC-AA) was then taken for further HPLC-UV analysis.

A Venusil-AA amino acid analytical column (4.6 mm ×250 mm, 5 μm) was used with a mobile phase of 80% acetonitrile-water (A) and 7.6% aqueous sodium acetate (pH =6.5) + 7% acetonitrile (B). The separation used a gradient elution with the following conditions: 0–10 min, isocratic 100% A; 10–15 min, linear gradient 100–90% A; 15–25 min, linear gradient 90–70% A; 25–33 min, linear gradient 70–55% A; 33.1–38 min, isocratic 0% A; and 38.1–45 min, isocratic 100% A. The flow rate was 0.8 mL/min, with a column temperature of 40 °C, an injection volume of 10 μL, and the detected wavelength of 254 nm.

## Results and discussion

### Alteration of weight and survival rates in DEN-induced HCC rats with or without 1,4-GL treatment

During 1,4-GL intervention, the rats in the control group eat well and have a stable mental status. By contrast, the rats in the DEN group have a gradual decrease in diet and activity. The body weights of DEN + 1,4-GL group were slightly higher than that of the DEN group. The variations in the weights of rats in the experiment are shown in Table 1.

**Table 1.** Alteration of rat weight before and after DEN treatment (Mean ± S.E.).

Group	Sex	Weight before treatment (g)	Weight on the 20th week, (g)
Control group	Female	206.25 ± 7.2 (*n* = 5)	330.23 ± 12.4 (*n* = 5)
	Male	220.62 ± 12.5 ( *n* = 5)	505.53 ± 22.1 (*n* = 5)
DEN group	Female	216.30 ± 8.3 (*n* = 10)	170.23 ± 13.2 (*n* = 3)
	Male	217.48 ± 13.4 (*n* = 10)	306.60 ± 15.5 (*n* = 6)
DEN + 1,4-GL group	Female	119.00 ± 11.6 (*n* = 13)	213.17 ± 10.6 (*n* = 8)
	Male	208.11 ± 11.2 (*n* = 13)	360.36 ± 13.6 (*n* = 10)

The rats in the control group did not die, and no evidence of liver cancer and liver disease was found, whereas the rats in the DEN and DEN + 1,4-GL group died during the experiment. The survival rate of each group of rats is shown in Table 2. The liver samples of the surviving rats of the DEN and DEN + 1,4-GL groups were confirmed to have liver cancer by pathological examination, showing a success rate of 100% cancer induction. Our experimental results show that 1,4-GL can improve the survival rate of DEN-induced HCC rats (from 45 to 70%).

**Table 2.** Survival and cancer induction rate of DEN-induced HCC rats.

Group	Sex	Number of rats (before treatment)	Number of surviving rats (20th week )	Survival rate (%)	HCC induction rate (%)
Control group	Female	5	5	100	0
	Male	5	5		
DEN group	Female	10	3	45	100
	Male	10	6		
DEN + 1,4-GL group	Female	13	8	70	100
	Male	13	10		

### Hepatic pathological changes in DEN-induced HCC rats with or without 1,4-GL treatment

**Figure 1.**
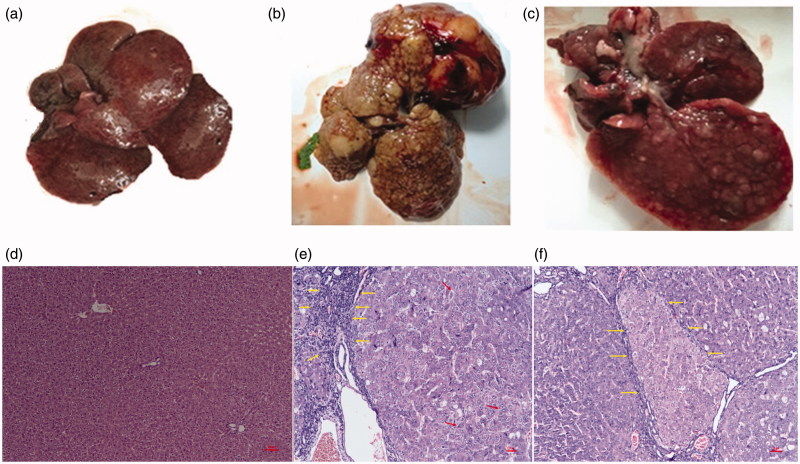
Photographs of the livers of healthy (a), DEN-induced HCC rat (b) and HCC rat administered 1,4-GL (c); Hepatic pathological changes (HE ×100) in healthy rat (d), DEN-induced HCC rat (e), and HCC rat administered 1,4-GL (f).

The liver morphology of the rats in the DEN and DEN + 1,4-GL group at 20 weeks is shown in Figure 1(a–c). As shown in the figure, the degree of hepatic deterioration in the DEN + 1,4-GL group was slower than that in the DEN group, and the volume of cancerous nodules and tumour decreased.

The liver pathological examinations of the DEN and DEN + 1,4-GL groups are shown in Figure 1(e,f). The figures show that the DEN rats lost the normal structure of their liver (Figure 1(e)) and showed many abnormal cells with irregular nuclear shapes (arrows). The liver deposits presented a large amount of fibrous tissue (Yellow arrow). In the DEN + 1,4-GL group, the liver cell morphological structure was still poor and separated by fibrous connective tissue. However, the DEN + 1,4-GL group had slightly slender fibrous connective tissue (arrows; Figure 1(f)), lesser invasive cancer cells, and slightly better cell atypia than the DEN group. The results showed the positive effect of 1,4-GL on liver cancer.

### The serum AFP contents in rats

The serum AFP was 10.26 ± 2.35 ng/mL (*n* = 10) in healthy rats and 18.56 ± 4.65 ng/mL (*n* = 9) in DEN-induced HCC rats, which was significantly higher (*p* < 0.01, ANOVA). However, the DEN + 1,4-GL group had a serum AFP of 14.28 ± 2.89 ng/mL (*n* = 18), which was significantly lower than that of the DEN group (*p* = 0.012, ANOVA), indicating that 1,4-GL decreased the degree of cancer induced by DEN.

### Multivariate analysis of ^1^HNMR spectra

**Figure 2.**
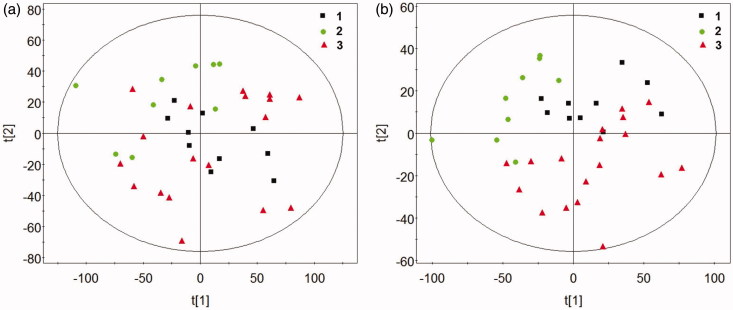
The Scores plot of PCA (a) and PLS-DA (b) by ^1^HNMR spectra of rat serum (1: Control group, 2: DEN group, 3: DEN + 1,4-GL group).

The results of ^1^HNMR spectra of the control, DEN and DEN + 1,4-GL groups analyzed by PCA and PLS-DA are shown in Figure 2. As shown in the figure, the samples in each group gathered in the same area, indicating that the differences between the groups were greater than those within the group. The control group was significantly separated from the DEN group. Moreover, the samples in the DEN + 1,4-GL group was closer to the control group than to the DEN group, indicating that 1,4-GL intervention might be contributing to the restoration of serum metabolite levels in HCC rats.

### Differential serum metabolite among three groups

Based on the PLS-DA model, the metabolites with the variable importance (VIP) values > l.0 were considered contributing to the classification. Twelve metabolites were identified and their integral peak areas were statistically analyzed in the study (Table 3). Compared with the control group, serum levels of acetate, glutamate, tyrosine and formate were increased in the DEN group, whereas those of leucine, isoleucine, valine, 3-hydroxybutyrate, lactate, glutamine, citrate and glycine serum were decreased. Whereas, in the serum of DEN + 1,4-GL group, the levels of BCAA (such as leucine and valine); energy metabolites (such as 3-hydroxybutyrate, lactic acid and acetic acid); and glutamine were apparently restored.

**Table 3.** Information of rat serum metabolites for classification observed by ^1^HNMR spectra.

Metabolite	δ^1^H	VIP	Integral interval	Relative levels in serum (mean ± SE.)		
				Control Group	DEN Group (*n* =	DEN + 1,4GL Group
				(*n* = 10)	9)	(*n* = 18)
Leucine	0.94 (d)	2.28	0.95–0.98	2.97 ± 0.01	2.87 ± 0.02*	2.90 ± 0.03
Isoleucine	1.02 (d)	2.15	0.98–1.00	2.58 ± 0.02	2.48 ± 0.02*	2.50 ± 0.02
Valine	1.04 (d)	2.41	1.03–1.05	2.79 ± 0.01	2.65 ± 0.01*	2.70 ± 0.02#
3-Hydroxybutyrate	1.20 (dd)	3.01	1.19–1.22	3.30 ± 0.03	2.89 ± 0.02*	3.09 ± 0.04#
Lactate	1.32 (d)	2.67	1.32–1.35	4.54 ± 0.03	4.06 ± 0.03*	4.31 ± 0.03#
Acetate	1.91 (s)	2.30	1.92–1.93	2.39 ± 0.02	2.59 ± 0.03*	2.44 ± 0.02#
Glutamate	2.34 (m)	1.89	2.31–2.32	3.15 ± 0.03	3.43 ± 0.03*	3.40 ± 0.03
Glutamine	2.44 (m)	2.27	2.44–2.46	2.53 ± 0.02	2.35 ± 0.03*	2.45 ± 0.02#
Citrate	2.55 (d)	2.81	2.64–2.66	2.08 ± 0.03	2.16 ± 0.03*	2.21 ± 0.02#
	2.65 (d)					
Glycine	3.57 (s)	2.21	3.56–3.58	2.69 ± 0.02	2.66 ± 0.02*	2.78 ± 0.02#
Tyrosine	6.88 (dd)	2.10	6.89–6.92	2.88 ± 0.02	2.96 ± 0.04*	2.99 ± 0.04
	7.18 (dd)					
Formate	8.45 (s)	1.55	8.45–8.47	2.03 ± 0.03	2.14 ± 0.02*	2.10 ± 0.03

VIP: variable importance for classification.

* Significantly different (*p* < 0.05) from the Control group.

# Significantly different (*p* < 0.05) from the DEN group.

### Elevation of Fischer’s ratio in the serum of HCC rats administered with 1,4-GL

The liver is an important organ that enables AA metabolism. Metabolic disturbance of AAs would appear in the body when the liver is damaged. Fischer’s ratio is commonly used as a clinical indicator of liver damage and dysfunction (Ishikawa 2012). It was calculated as the ratio of BCAAs (serum contents of leucine, isoleucine and valine) to aromatic AAs (serum contents of phenylalanine and tyrosine).

**Figure 3.**
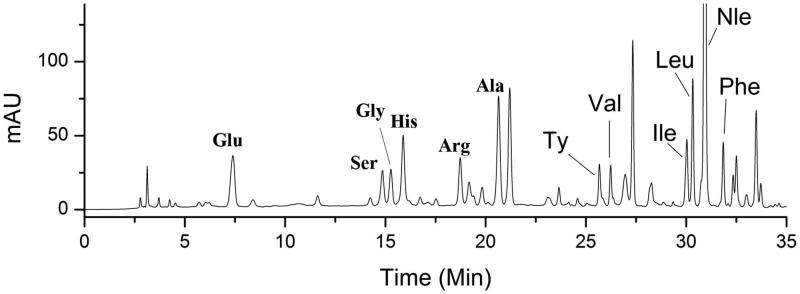
Typical HPLC-UV chromatogram (254 nm) of rat serum after PITC derivatization. Glu: glutamate; Ser: serine; Gly: glycine; His: histidine; Arg: arginine; Ala: alanine; Ty: tyrosine; Val: valine; Ile: isoleucine; Leu: leucine; Nle: norleucine; Phe: Phenylalanine; PITC: phenylisothiocyanate.

Amino acid – PITC derivatives are stable with high sensitivity of UV detection (254 nm), though some limitations exist such as high toxicity of PITC and the reduced service life of the chromatographic column. Therefore, an HPLC-UV method was employed to determine AAs in rat serum with PITC derivatization in this study. The normal range of Fischer’s ratio is between 3.0 and 4.2 in healthy individuals, but in the presence of liver diseases, the ratio declines in proportion to the severity of the liver damage (Campollo et al. 1992; Ishikawa 2012). The rat serum AA chromatogram is shown in Figure 3. Our results suggested that Fischer’s ratio in the serum of healthy rats was 2.3 ± 0.12, a significant decrease of that observed in HCC rats (1.62 ± 0.21). However, Fischer’s ratio increased to 1.89 ± 0.22 when the HCC rats were orally administered 1,4-GL. A prospective cohort study showed that Fischer’s ratio has an inverse association with HCC risk (Stepien et al. 2016). Furthermore, Tsuchiya’s study proposed that the long-term oral administration of BCAA particles in HCC patients treated with radiotherapy can reduce the recurrence rate of HCC and improve the survival rate of patients with an albumin less than 35 g/L (Muto et al. 2006). Therefore, our results further demonstrate that 1,4-GL exhibits some protective effect against DEN-induced HCC.

## Conclusions

1,4-GL is naturally found in many vegetables, fruits, and classic Chinese medicine LWPs. Until now, no research using metabonomics investigates the role of 1,4-GL in preventing liver cancer. The results of this study showed that the survival rate of DEN-induced HCC rats increased from 45% to 70% when orally treated with 1,4-GL. The pathological results showed that 1,4-GL exerted positive effects on liver carcinogenesis. Compared with that in the DEN group, the serum AFP level in the DEN + 1,4-GL group significantly decreased. The ^1^HNMR-based metabonomic analysis revealed that the serum levels of BCAA, 3-hydroxybutyrate, lactate, acetate and glutamine in the DEN + 1,4-GL group tended to normalize compared with the DEN group. Furthermore, the Fischer’s ratio of the DEN + 1,4-GL group significantly increased. The present study further confirmed that 1,4-GL has certain pharmacological effects on the prevention of HCC induced by DEN, and its mechanism of action may be related to the restoration of AAs and energy metabolism.
